# Investigation of Size and Morphology of Chitosan Nanoparticles Used in Drug Delivery System Employing Chemometric Technique

**Published:** 2015

**Authors:** Mohammadreza Khanmohammadi, Hamideh Elmizadeh, Keyvan Ghasemi

**Affiliations:** *Department of Chemistry, Faculty of Science, International University of Imam Khomeini, Qazvin, Iran.*

**Keywords:** Chitosan nanoparticles, DRIFT spectroscopy, Chemometrics, Particle size

## Abstract

The polymeric nanoparticles are prepared from biocompatible polymers in size between 10-1000 nm. Chitosan is a biocompatible polymer that - can be utilized as drug delivery systems. In this study, chitosan nanoparticles were synthesized using an optimized spontaneous emulsification method. Determining particle size and morphology are two critical parameters in nanotechnology. The aim of this study is to introduce methodology based on relation between particle size and diffuse reflectance infrared fourier transform (DRIFT) spectroscopy technique. Partial least squares (PLS) technique was used to estimate the average particle size based on DRIFT spectra. Forty two different chitosan nanoparticle samples with different particle sizes were analyzed using DRIFT spectrometry and the obtained data were processed by PLS. Results obtained from the real samples were compared to those obtained using field emission scanning electron microscope(FE-SEM) as a reference method. It was observed that PLS could correctly predict the average particle size of synthesized sample. Nanoparticles and their morphological state were determined by FE-SEM. Based on morphological characteristics analyzing with proposed method the samples were separated into two groups of "appropriate" and "inappropriate". Chemometrics methods such as principal component analysis, cluster analysis (CA) and linear discriminate analysis (LDA) were used to classify chitosan nanoparticles in terms of morphology. The percent of correctly classified samples using LDA were 100 %and 90% for training and test sets, respectively.

## Introduction

Chitosan is a linear polymer composed of *β*-(1–4)-2-amino-2-deoxy-D-glucopyranose units. Being a polycationic, nontoxic, biodegradable, non-immunogenic, antibacterial, and biocompatible polymer, chitosan has attracted much attention and has wide applications in biotechnology, pharmaceutical, textile, food, cosmetics, and agricultural industries (1-3). 

Tacrine (THA, 9-amino-1, 2, 3, 4-tetrahydroacridine), used in the treatment of Alzheimer’s disease, is known to induce hepatotoxicity, the mechanisms of which remain to be fully established (4).

Nanoparticles are defined as particulate dispersions or solid particles with a size in the range of 10-1000 nm (5). Today, nanotechnology is found in a wide range of applications in the pharmaceutical industry (6). Nanoparticles based on hydrophilic polymers, such as chitosan, are appropriate candidates for drug delivery (7-9) , because they have the advantage of prolonged circulation in the blood, which would facilitate extravasation and passive targetin (10). It is important to determine powdered pharmaceutical material particle size, since particle size distribution exerts a significant effect on the physical properties of the bulk material (11).

The polymeric nanoparticles are prepared from biocompatible polymers in size between 10-1000 nm. These can be accomplished by different methods such as: Solvent evaporation, Nanoprecipitation, Emulsification, Salting out and *etc* (12). Spontaneous emulsification obtained by the rapid diffusion of a water-soluble solvent, solubilized first in the oily phase, moving toward the aqueous one when the two phases are mixed (13,14).

Multivariate calibration methods have proven most successful in calibrating near infrared spectroscopy (NIRS) data to measure particle size. One recent application determined the median particle size of drugs by multiple linear regression, while Frake *et al.* used neural networks (15). Among chemometric methods, PLS frequently has been used because of the quality of the calibration models obtained, the ease of its implementation and the availability of software (16). DRIFT spectroscopy is a unique method for analyzing various opaque materials. The information obtained by DRIFT spectroscopy is governed by the size, shape, and surface morphology of the sample (17). Therefore, a direct comparison of materials with different properties in terms of the DRIFT spectra is generally difficult. In many studies, methods based on DRIFT spectroscopy in combination with chemometric techniques have been used as a quick, novel and efficient technique for qualitative and quantitative analysis (18-20). Different chemometric methods such as principal component analysis (PCA), CA and LDA were used to classify the samples (21-23).The potential application of infrared spectroscopy was suggested for determining particle size of powders (25).

This study investigates the relationship between morphology and size of chitosan nanoparticles and DRIFT spectroscopy. Chitosan nanoparticles were synthesized according to the Box-Behnken response surface methodology to achieve the minimum size and suitable morphology of nanoparticles. Ultraviolet spectrometer at 240 nm was applied to determine the drug loading capacity (25). Also, Morphological properties were assessed with FE-SEM. Different chitosan nanoparticles with different morphologies were analyzed by DRIFT spectroscopy and the obtained data were processed by multivariate classification methods. In this work, different unsupervised pattern recognition techniques, such as PCA, CA and supervised techniques, such as LDA, were applied. Also, another aim of this work was to devise a procedure for DRIFT calibrations to measure the average particle size of chitosan nanoparticles using all the data from the reference (FE-SEM) method in one mathematical step using partial least squares regression. Further, in this research, PLS was applied to propose a rapid and reliable approach for estimation of the average size of chitosan nanoparticles.

## Experimental

*Materials*

The drug tacrine (9-amino-1, 2, 3, 4-tetrahydroacridine), Glutaraldehyde solution and Span 80 were from Sigma (St. Louis, Missouri). Linseed oil was from Aldrich. Chitosan was from Suvchem (India). Nano Fe_3_O_4_ with particle size of 30 nm was from Neutrino Company (China). All other chemicals and reagents used were of analytical grade.

*Instruments and software*

The particle size and morphology of synthesized nano materials were determined using a field emission scanning electron microscope (FE-SEM, 15 kV, model 54160, Hitachi, Japan). The UV-Vis spectrophotometer was from Camspec Co. (model, M350). UV-Vis spectra were recorded in 210-400 nm spectral region with a data point spacing of 1 nm. 

Mid-infrared spectrometer (Nicolet, Madison, WI, USA) equipped with diffuse reflection sampling cell with a DTGS detector and a CsI beam splitter was employed. FT-IR spectra were recorded in 900-4000 cm^-1^ spectral region with a data point spacing of 3.85 cm^-1^. For digitization of the spectra, WINFIRST software version 3.57 was used. Sizes of nanoparticles from FE-SEM images were determined by Microstructure Measurement software called Nahamin Pardazan Asia version 1.0. This software uses image processing technology to estimate the average size of nanoparticles according to the captured image. Pls-Plus/Iq button to GRAMS/32 and higher software (GalacticInd. Co.) were used to process the absorbance data (26,27).

*Preparation of Chitosan Nanoparticles Samples*

Chitosan (100 mg) and required quantities of sodium chloride (as an electrolyte) were dispersed in glacial acetic acid (3 %, 20 mL) and stirred continuously for 2 hours to obtain chitosan gel. Then the solution was kept overnight to obtain clear chitosan gel. In the next step, chitosan gel (5 mL) was added dropwise into linseed oil (10 mL) containing required quantities of Span 80 as a surfactant under magnetic stirring for 30 minutes at room temperature. Then acetone (5 mL) was added drop by drop (2 mL/min) with a micropipette to the system. The system was maintained under stirring for 1 hour while covered in aluminum foil, and then, the beaker was kept open to let the polymer precipitate through evaporation of acetone with subsequent formation of smaller spheres suspended in oil phase. Then required quantities of Glutaraldehyde-Saturated Toluene (GST) as a chemical cross-linking agent were added to the system slowly and stirred for 2 hours continuously to solidify and stabilize the spheres. The nanoparticle suspension was obtained with centrifugation at 5000 rpm for 30 minutes, being washed three times, each time with toluene and acetone. Finally the product was dried at room temperature to obtain a free-flowing, fine powder. Since nanoparticle size strongly depends on synthesis parameters such as sodium chloride, surfactant and chemical cross-linking agent, it was possible to synthesize different samples by changing these parameters. this method was optimized using design of experiments (DOE) by employing a 3-factor, 3-level Box–Behnken statistical design. Box–Behnken experimental design as a response surface method can be used to evaluate the relationship between the studied parameters and their effects on the size of chitosan nanoparticles. Three factors (Span 80 (2–10 V/V %), NaCl (0.5–2.5 W/W %), and glutaraldehyde saturated toluene (GST) (2–6 mL), 3-level Box–Behnken design on the measured response (particle size) were established for this optimization procedure (25).

*Preparation of chitosan nanoparticles containing tacrine drug*

Chitosan nanoparticles containing tacrine were prepared using the optimal synthesis method provided by the experimental design (25). Chitosan gel (5 mL) was taken in a beaker (100 mL) and the drug tacrine was dissolved in chitosan gel (drug-to-polymer ratio of 1:1(w/w)) under magnetic stirring. Then, this gel was added dropwise into linseed oil (10 mL), which contained optimum quantity of Span 80, under magnetic stirring for 30 minutes at room temperature. Then, acetone (5 mL) was added drop by drop (2 mL/min) with a micropipette. The other steps of synthesis process were in accordance with the preparation of chitosan nanoparticles.

## Results and Discussion

*Particle size analysis*

**Figure 1:**
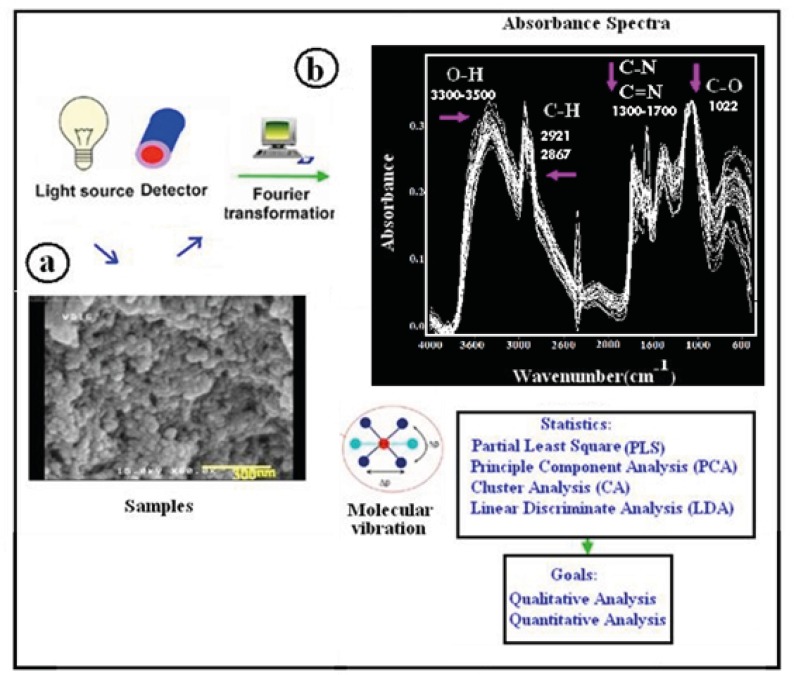
Analytical workflow in DRIFT spectroscopy

As mentioned before, all the samples were studied using FE-SEM to determine their average particle size and morphology. FE-SEM from the external surface of chitosan nanoparticle samples provided the possibility to observe the structural situation. The particle size of the final product was clearly observed to depend on designed parameters during the synthesis procedure. The designed nanoparticles have average particle sizes from 33.64 to 74.87 nm, which were determined by FE-SEM (Figure 1 (a)).

*Determining the tacrine content in chitosan nanoparticles*

There are two methods for drug loading in micro/nanoparticulate systems: during the preparation of particles (incorporation), and after the formation of particles (incubation). In these systems, drug is physically embedded into the matrix or adsorbed onto the surface (2). Yield percentage and drug loading percentage of chitosan nanoparticles that were synthesized according to the optimum method were 90 % and 13.4 ± 0.51 %, respectively (25). 

*DRIFT analysis*

DRIFT spectroscopy is a powerful tool for the study of materials such as polymers and powders. The bulk or surface morphology of these materials is many times an important experimental parameter that can be altered by sample preparation methods used in the more common spectroscopic techniques. DRIFT spectroscopy has been shown to be more sensitive to surface species than transmission measurements and to be an excellent in situ technique. Vibrational spectra of nanoparticles generally differ from those of respective bulk materials due to quantum confinement effect and size effect (28), and surface amorphousness so an important conclusion is that the optical absorption and scattering in the fundamental lattice absorption region should be size-dependent. The frequency shift and the shape of the vibrational spectra also depend on the surface morphology of the materials (29). DRIFT analyses of synthesized chitosan nanoparticles are shown in Figure 1 (b). DRIFT spectra were taken from synthesized chitosan nanoparticle samples. Chitosan nanoparticle spectrum has free amine, hydroxyl and ether functional group. In the spectra, the strong and wide peak in the 3500-3300 area is attributed to hydrogen-bonded O-H stretching vibration. Also, the peaks at 2921 cm^-1^ and 2867 cm^-1^ correspond to the stretching vibrations of C-H .The stretching vibrations of C-O are found at 1088 cm^-1^ and 1022 cm^-1^ (30). Amino groups are located at 1300-1700 cm^-1^. The applied analytical strategy is shown in MIR workflow depicted in Figure 1.The sample is measured in diffuse reflection; functional groups are excited by stretching and bending vibrations and absorbed energy is recorded. The intensity of light reflected is sent towards a DTGS detector and spectrum is generated by fourier transformation (FT). As differences in spectra resulting from morphology differences are in some cases hard to interpret, chemometric algorithms including PCA, CA, LDA and PLS are applied. By this, qualitative and quantitative analysis can be achieved. 

*Quantitative analysis*

Particle size is routinely determined by FE-SEM; for the establishment of a DIRIFT-model, particle diameters provided by individual manufacturers were taken for calibration. 42 spectra of 15 different chitosan nanoparticles were recorded in a wave number range of 900-4000 cm^-1^ in diffuse reflection mode. PLS is always an important tool when there is partial knowledge of the data. PLS can be very robust provided that future samples contain similar features to the original data, but the predictions are essentially statistical. Multivariate calibrations are useful tools to be used in spectral analysis in order to overcome the spectral overlapping and to improve the precision and the predictive ability of the FT-IR spectrometry. With the aim of quantitative analysis of chitosan nanoparticles, the PLS multivariate model was applied with the absorption spectra data. Spectral information was mean centered prior to PLS data treatment. The net analyte signals (NAS) are useful measures in building and optimizing multivariate calibration models. Also, in this research, PLS-NAS model was applied to propose a rapid and reliable approach for estimation of the average size of chitosan nanoparticles.

**Figure 2:**
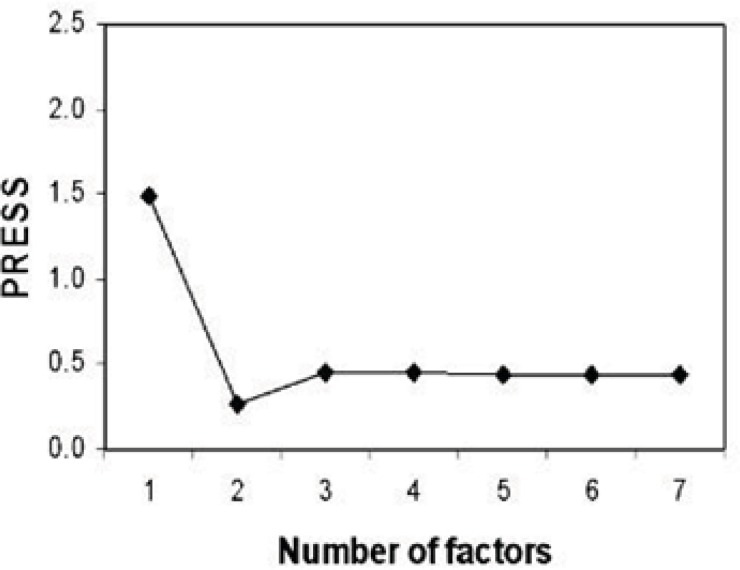
Diagram of PRESS according to number of factors for PLS–NAS model.

**Figure 3:**
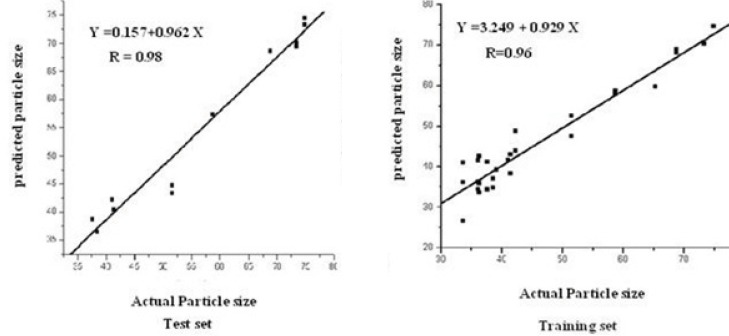
Graphical analysis of the predictive ability of PLS–NAS on the independent test set.

Determining the optimum number of factors (rank) to be used in the calibration is a key step in PLS. The predicted residual error sum of squares (PRESS) was used to determine the optimum number of factors in PLS. Figure 2 shows the calculated PRESS during the variation of the number of factors, contributed in the model. However, the optimum number of factors was found to be 2 for calibration set. Training set and test set samples were selected using Kennard Stone sampling algorithm. For training studies, 29 samples were selected, and the other samples from 13 synthesized chitosan nanoparticles were selected for verification. Figure of merit for 13 samples of test sets is illustrated in Table 1. The root mean square error (RMSE) and the squared correlation coefficient of regression lines (R^2^) for test set samples were also calculated (Table 1). The predictive abilities of PLS-NAS model on the independent test set are shown in Figure 3. Even the prediction of particle size in chitosan nanoparticles showed high linearity. 

**Table 1:** Actual and predicted mean particle size of chitosan nanoparticles samples with PLS-NAS algorithm for independent test set.

Sample	Actual	Predicted by PLS-NAS
**1**	41	42.18
**2**	41	42.12
**3**	74.87	73.29
**4**	74.87	74.44
**5**	51.52	43.36
**6**	51.52	44.76
**7**	37.6	38.70
**8**	68.72	68.65
**9**	73.33	69.40
**10**	73.33	70.20
**11**	58.71	57.35
**12**	41.38	40.39
**13**	38.5	36.44
a **RMSE**		3.59
**R** ^2^		0.98

a Root Mean Square Error.

**Figure 4:**
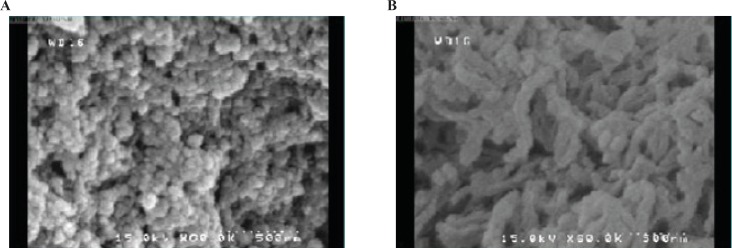
FE-SEM images of optimized chitosan nanoparticles with appropriate morphology (a), chitosan nanoparticles with poor morphology (b).

In this work, the prepared chitosan nanoparticles were analyzed by FE-SEM in terms of size and morphology. The images were classified into two groups of appropriate and inappropriate morphology (Figure 4). Appropriate morphology is related to the particles which have spherical shapes and uniform distributions. Classification techniques were used for qualitative analysis of synthetic nanoparticles. Size and morphology of the polymer matrix are assumed to have an extremely important role in the drug release and pharmacokinetics (31). Morphological classification of synthetic nanoparticles was done to test if the DRIFT spectroscopy was an applicable method for determining morphological structure of nanoparticles otherwise determined using the FE-SEM. About 42 DRIFT spectra were taken from the produced chitosan nanoparticles. 

*Principal components analysis*

**Figure 5:**
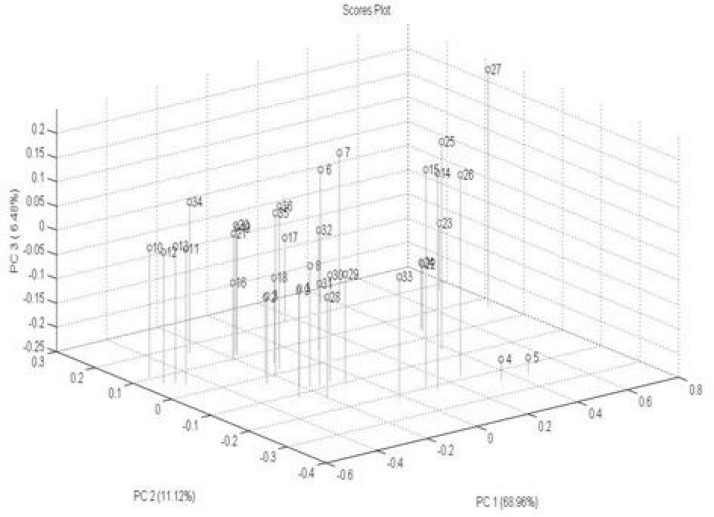
Score plot of first 3 PC for separating spectral data.

**Figure 6:**
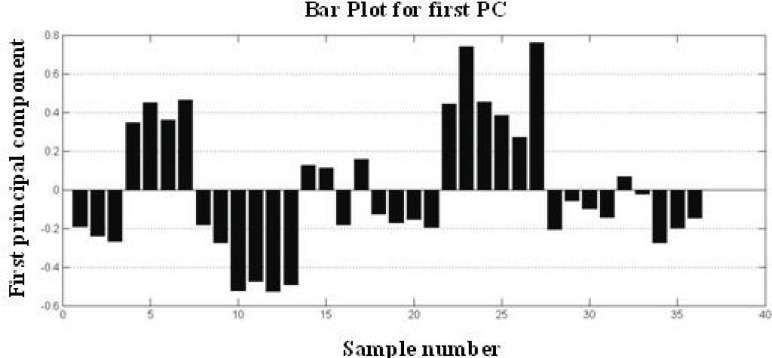
Bar Plot of first score for visualizing role classification power of PCA.

PCA is a standard tool in chemometrics for that uses an orthogonal transformation to convert a set of observations of possibly correlated variables into a set of values of linearly uncorrelated variables called principal components. It is a way of identifying patterns in data, and expressing the data in such a way as to highlight their similarities and differences. PCA implies a mathematical procedure that transforms the overall set of original variables into smaller numbers of mathematical "constructs". Such constructs can be easily viewed as linear combinations of the original variables (32). A total of 36 DRIFT spectra were processed by PCA and scores were extracted. Plotting these scores, Figure 5 shows that 86.56 % of the total variance of data is carried by the first 3 PC. The variances are about 68.56 %, 11.12 % and 6.48 % for PC1, PC2 and PC3, respectively. One method for evaluating how much is successfully of PCA is to plot score plot that describe the under study samples in maximum variance direction Figure 5. According to the figure of score plot, there is not a clear visual separation line between the samples. The bar plot of the first PC values is shown in Figure 6. The bar plot is another way for determining the success of the principal component analysis technique. In this method, all samples of a same class are located on one side of the chart, and samples of other groups are on the opposite side. As shown in figures 5 and 6, sample interferences are relatively high. In other words, it can be stated that this method is weak for the morphological classification of chitosan nanoparticles.

*Cluster analysis*

**Figure 7:**
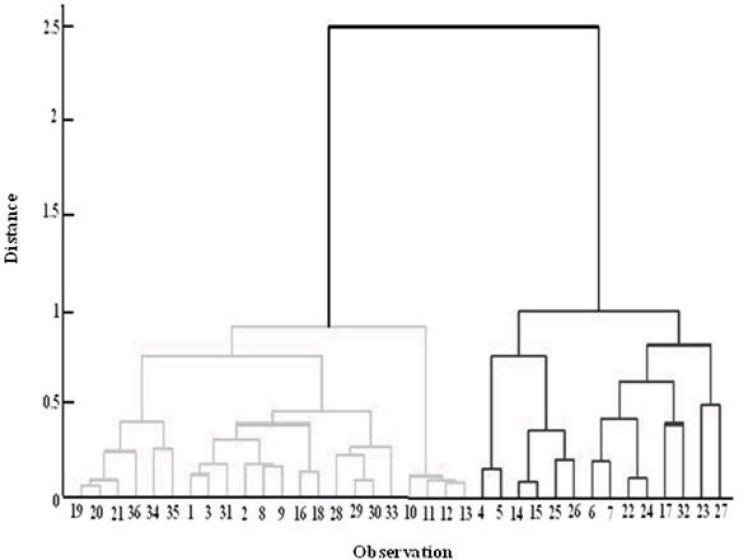
Dendrogram of cluster analysis according to ward distance calculation.

Cluster analysis assesses the similarity between samples by measuring the distances between the points in the measurement space. Samples that are similar lie close to one another, whereas dissimilar samples are distant from each other. The choice of the distance metric to express similarity between samples in a data set depends on the type of measurement variables used. Measurement variables are usually continuous. Ward Distance was used for cluster analysis of the data. This technique exhibited a good performance for the separation of samples into two classes. Figure 7 shows the dendrogram of this analysis. Although samples are divided into two distinct groups according to entering distance, they are not matched with two other groups; for example, 8 and 9 are in the inappropriate class but they are located in the appropriate class of morphology. This shows that CA as a classification method could not separate these two samples correctly.

*Linear Discriminant Analysis (LDA)*

Supervised multivariate methods, such as LDA, are powerful tools to build rules of discrimination used later to identify new samples. LDA searches for the variables containing the greatest inter-class variance and the smallest intra-class variance and constructs a linear combination of the variables to discriminate between the classes. The rule is constructed with the training set of samples and further tested with the test set. In this research, we tried to study the morphology of chitosan nanoparticles using DRIFT spectroscopy, proposing a treatment of the results by LDA in order to improve the reliability of data interpretation. The linear combination for a discriminant analysis is derived from Equation (1), as:

Z = w_1 _X_1 _+ w_2 _X_2 _+ w_3 _X_3 _+ …+ w_n _X_n                     _Equation (1)

Where Z is the discriminant score, w_i_ is the discriminant weight for independent variable *i* and X_i_ is the independent variable *i*. In this case, the independent variable corresponded to the absorbance in a wavenumber (18). Each w_i_ was set so as to maximize the between-group variance of Z (Z variance between poor (bad) morphology group of chitosan nanoparticles and good morphology group of chitosan nanoparticles) relative to the within-group variance of Z (Z variance within bad morphology group of chitosan nanoparticles and good morphology group of chitosan nanoparticles). 

Training set and test set samples were selected using Kennard Stone sampling algorithm. For training studies, 15 samples were selected, and the other samples from 36 synthesized chitosan nanoparticles were selected for verification. Figure of merit for training and test sets are illustrated in Table 2(b). LDA technique was successful in distinguishing the morphology of nanoparticles that are shown in Table 2(a). The results of the predicted classes for test set indicate that LDA is a powerful methodology for classification of DRIFT spectra and could be used for nanoparticle characterization.

**Table 2:** a) Classification power of LDA for test set. (b) Figure of merit for training and test set.

^(a)^Sample	Actual class	Prediction class	Convergence rate of the class with appropriate morphology.(1)	Convergence rate of the class with poor morphology.(2)
1	1.00	1.00	0.77	0.23
2	1.00	1.00	1.00	0.00
3	1.00	1.00	0.96	0.04
4	2.00	2.00	0.00	1.00
5	1.00	1.00	1.00	0.00
6	1.00	1.00	1.00	0.00
7	1.00	1.00	1.00	0.00
8	1.00	1.00	1.00	0.00
9	1.00	1.00	1.00	0.00
10	1.00	1.00	1.00	0.00
11	1.00	1.00	1.00	0.00
12	2.00	2.00	0.00	1.00
13	2.00	1.00	1.00	0.00
14	2.00	1.00	1.00	0.00
15	2.00	2.00	0.00	1.00
16	2.00	2.00	0.00	1.00
17	1.00	1.00	1.00	0.00
18	1.00	1.00	1.00	0.00
19	1.00	1.00	1.00	0.00
20	1.00	1.00	1.00	0.00
21	1.00	1.00	1.00	0.00
				
^(b)^Parameter	Test Set	Training Set	Definition	
Correct rate:	0.9048	1	Correctly Classified Samples /Classified Samples	
Error rate:	0.0952	0	Incorrectly Classified Samples /Classified Samples	
Sensitivity samples:	1	1	Correctly Classified Positive Samples / True Positive	
Specificity samples:	0.6667	1	Correctly Classified Negative Samples /True Negative	

## Conclusions

The Preparation of chitosan nanoparticles as delivery systems for the anti-Alzheimer drug tacrine was the goal of this study. Also, nano-carrier size is very important in drug delivery. Therefore, the findings suggest that the analysis of nanoparticle sample by DRIFT spectrometry and data processing by PLS-NAS would provide a novel method for average nanoparticle size estimation. The results point out high suitability of DRIFT spectrometry to be used in the field of nano-technology for quantitative investigations in material science. The established PLS-NAS model is characterized by high linearity, precision, reproducibility and speed of analysis. Also, DRIFT spectrum of chitosan nanoparticles was coupled with chemometric techniques for qualitative analysis. The findings suggest that the analysis of chitosan nanoparticles samples by DRIFT spectroscopy and data processing by multivariate classification methods would provide a novel method for determining chitosan nanoparticles morphology. LDA technique was successful in morphological classification of chitosan nanoparticles samples.
